# Development and Validation of the LC–MS/MS Method for Determination of 130 Natural and Synthetic Cannabinoids in Cannabis Oil

**DOI:** 10.3390/molecules27238601

**Published:** 2022-12-06

**Authors:** Natalia Galant, Jakub Czarny, Jolanta Powierska-Czarny, Agnieszka Piotrowska-Cyplik

**Affiliations:** 1 Institute of Forensic Genetics, Al. Mickiewicza 3/4, 85-071 Bydgoszcz, Poland; 2 Department of Food Technology of Plant Origin, Poznan University of Life Sciences, Wojska Polskiego 31, 60-624 Poznan, Poland

**Keywords:** cannabinoids, cannabis oil, dietary supplements, LC–MS/MS

## Abstract

Dietary supplements are widely available products used by millions of people around the world. Unfortunately, the procedure of adding pharmaceutical and psychoactive substances has recently been observed, in order to increase the effectiveness of supplements in the form of hemp oils. For this reason, it is extremely important to develop analytical methods for the detection of substances prohibited in dietary supplements and food products. In the present study, using the LC–MS/MS technique, an innovative method for the detection and quantification of 117 synthetic cannabinoids and 13 natural cannabinoids in dietary supplements and food products in the form of oils during one 13-min chromatographic run was developed. Each method was fully validated by characterization of the following parameters: The limit of detection was set to 0.1 ng/mL (100 µg/g, 0.01%). The limit of quantification ranged from 0.05 ng/mL to 50 ng/mL. The criteria assumed for systematic error caused by methodological bias (±20%) resulting from the recovery of analytes after the extraction process, as well as the coefficient of variation (CV) (≤20%), were met for all 130 tested compounds. The positive results of the validation confirmed that the developed methods met the requirements related to the adequacy of their application in a given scope. Additionally, methods developed using the LC–MS/MS technique were verified via proficiency tests. The developed analytical procedure was successfully used in the analysis of hemp oils and capsules containing them in the studied dietary supplements.

## 1. Introduction

The growing public awareness of health has caused an increasing proportion of the population to be interested in nutritional supplements. Manufacturers offer a ready-made solution in the form of dietary supplements for this group of consumers [1,2]. Their use has become a common and fashionable practice not only in North America, but also in Asia and Europe [3,4]. Unfortunately, counterfeiting is a constantly growing problem in the dietary supplement sector. A commonly used strategy for counterfeiting dietary supplements is adding undeclared pharmaceutical substances (often withdrawn from use due to their negative effects), as well as their analogues [5,6,7].

Due to its wide range of analytical possibilities, mass spectrometry is the leading method used in the research of pharmaceutical products and dietary supplements.

In combination with gas chromatography (GC) or liquid chromatography (LC), mass spectrometry finds a number of applications in controlling the quality and safety of dietary supplements. The extensive literature on the subject confirms that conjugate techniques are also perfect for detecting, confirming, and quantifying pharmaceuticals and psychoactive substances, as well as for studying the structures of new compounds added to this type of product [8,9,10].

Due to their high sensitivity and selectivity, tandem mass spectrometers are very popular. In studies of dietary supplements, these types of spectrometers are often combined with a liquid chromatograph (LC–MS/MS). They are frequently used for the analysis of dietary supplements supporting weight loss and for physically active people, usually in the form of stimulants and substances with anorexic, diuretic, or laxative effects [11,12,13,14].

Both single methods—e.g., Cohen et al. (2015) identified 1,3-dimethylbutylamine (DMBA) in dietary supplements using two identification variants: by using the MRM mode, and by comparing the retention time as well as the MS and MS/MS spectra with the reference substance [15]—and mixed methods [16,17,18] are being developed.

The analysis carried out in the MRM mode makes it possible to identify many compounds at the same time, without their complete chromatographic separation, provided that the selected ion transitions are specific for a given analyte, for practical and economic reasons. Bogusz et al. (2006) published a method of detecting the 80 most common synthetic admixtures (e.g., analgesics, antidiabetics, antiepileptics, psychotropics, anorectics, hormones, and anabolic drugs) in herbal preparations. For most analytes, two ion transitions for protonated or deprotonated pseudomolecular ions were monitored [19]. Recently, products made of hemp (*Cannabis sativa* L.)—which, unlike cannabis (*Cannabis indica*), is characterized by a low content of the psychoactive ∆9-tetrahydrocannabinol (THC)—have become very popular. The inflorescences and leaves of this plant species are rich in biologically active cannabinoids, including cannabidiol (CBD), which has many therapeutic properties [20,21,22,23]. The dietary supplement market has recently experienced a wave of CBD oils, which in addition to hemp seed oil include extracts containing cannabidiol and other cannabinoids that occur naturally in cannabis [24,25,26,27,28,29]. Much of the current research on cannabis products is based solely on the analysis of natural cannabinoids, focusing mainly on the determination of the total THC content (i.e., the sum of delta-9-tetrahydrocannabinol and delta-9-tetrahydrocannabinolic acid)—due to legal regulations regarding it—and CBD (i.e., the sum of cannabidiol and cannabidiolic acid), the presence of which is desirable due to its therapeutic effects. The use of gas and liquid chromatography with various types of detectors for the determination of natural cannabinoids is widely described in the literature [30,31]. There are also reports on the analysis of the composition of dietary supplements or food products in terms of their contents of synthetic cannabinoids. These compounds show toxic effects even at small concentrations [32].

A simple methanol extraction was used to isolate all test compounds from the matrix. In turn, Heo et al. [33] developed the LC–ESI–MS/MS method for the determination of natural and synthetic cannabinoids in food, herbs, and dietary supplements, in the form of capsules, tablets, liquids, cookies, and candies. The use of this technique in the analysis of cannabinoids in cannabis-based food products has also been described by some authors [34,35,36,37,38,39,40]. Research in the field of forensics demonstrates that these products most often appear on the market in the form of fortified plant materials that are indistinguishable from hemp for an average consumer. Therefore, there is a risk that these compounds—such as other psychoactive substances [41,42,43,44,45]—may be found in dietary supplements.

The improvement of the quality of dietary supplements largely depends on legal regulations. Increasing consumers’ safety by the means of increased control and intensified research on cannabis products entails the need for new analytical solutions. Therefore, a new LC–MS/MS method, allowing for the simultaneous determination and quantification of 13 natural and 117 synthetic cannabinoids present in the form of oil in dietary supplements and food products, was developed and validated.

## 2. Results and Discussion

### 2.1. LC–MS/MS Method

**Figure 1:**
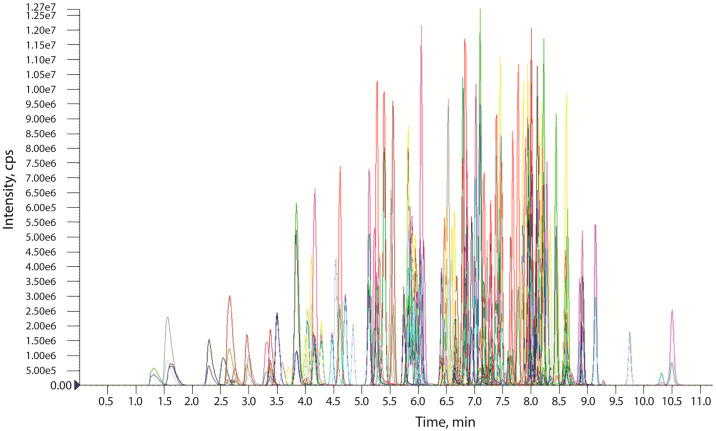
Extracted ion chromatogram of the working standard solution of 130 studied cannabinoids.

The developed LC–MS/MS method included two selective MRM transitions for 130 target analytes and one MRM transition for the internal standard. Despite the large number of substances analyzed, the detection was carried out a single 13-min chromatographic run (Figure 1). The detection windows of 40 s were sufficient for complete coverage of all of the chromatographic peaks. The minimum dwell time was 8.42 ms. Between 8 and 10 data points per peak were obtained for all analytes, ensuring good shape and reproducibility of the peaks. The ion transitions, MS parameters, and retention times for the analytes are shown in Table S1. Quantification was performed using the first transitions and it was selected because of the higher sensitivity.

### 2.2. Extraction

In this study, the extraction conditions of 130 cannabinoids from oil were optimized. Different extraction solvents, volumes, and extraction times were tested. Three real samples of cannabis oil and blank matrix samples spiked at low, medium, and high levels with the studied analytes were extracted under different conditions. The results indicated that increasing the volume of the extractant and extending the extraction time had no effect on the extraction recovery (Tables S2 and S3). Based on the results, extraction with 1 mL of acetonitrile and shaking for 10 min were chosen.

### 2.3. Method Validation

#### 2.3.1. Selectivity and Sensitivity

**Figure 2:**
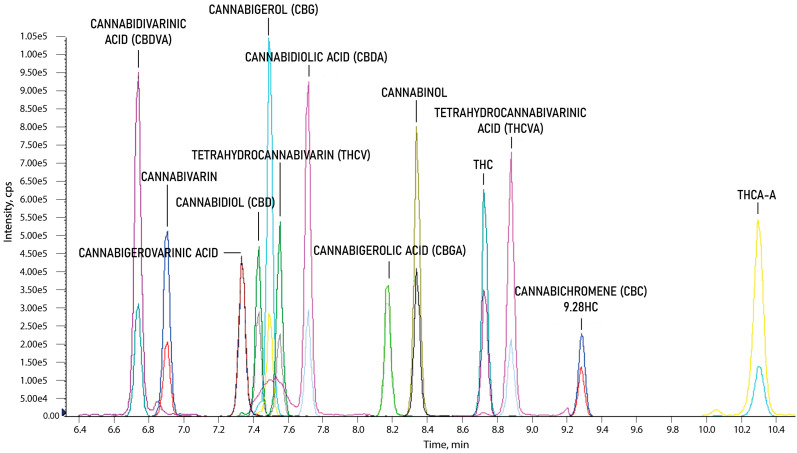
Chromatographic separation of 13 natural cannabinoids.

The selectivity of the developed method was investigated by comparing visible signals from two transition reactions for each analyte for the blank and spiked (with a mixture of all 130 standard solutions) oil samples. No significant interferences from the matrix with the retention time of the analyzed cannabinoids were observed. Chromatographic separation of analytes with different masses (±3 *m*/*z*) was not required using MS/MS methods, because of the specific MRM transitions for each compound. However, due to the simultaneous analysis of such a large number of similarly structured compounds (including isomers), coeluting for the same precursor mass (±2 *m*/*z* in the case of isotopic interferences) was checked. There was a high probability of breaking down into the same fragments via cell collisions and producing the same ion with a different intensity. This result could be the result of selecting the two most intense product ions characterized for more than one compound. Therefore, the chromatographic separations for all MRM pairs with differences in precursor mass of ±2 *m*/*z* were analyzed in detail. Chromatographic separation of all-natural cannabinoids—including structural isomers such as Group 1: THC, CBD, and CBC; Group 2: THCA-A and CBD; and Group 3: CBDVA and THCVA—was achieved by applying the developed gradient (Figure 2).

**Figure 3:**
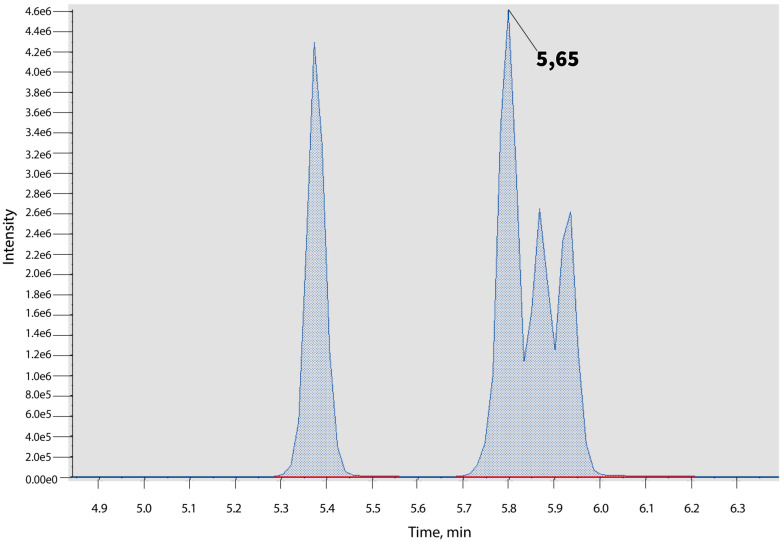
Unresolved chromatographic peaks for ADB-PINACA and its isomers.

From 119 synthetic cannabinoids, five compounds—ADB-PINACA and its four structural isomers (ADB-PINACA ISOMER 1, ADB-PINACA ISOMER 2, ADB-PINACA ISOMER 3, and ADB-PINACA ISOMER 4)—had the same MRM transition. There was one separated peak for ADB-PINACA ISOMER 1 (RT 5.42 min), and unresolved chromatographic peaks were observed for ADB-PINACA, ADB-PINACA ISOMER 2, ADB-PINACA ISOMER 3, and ADB-PINACA ISOMER 4 (Figure 3). Due to the fact that there were no other selective MRM pairs to choose and no chromatographic separation for these cannabinoids in the applied gradient, quantitation analyzes were performed for all of these compounds.

The limit of quantification (LOQ) was determined as 0.1 ng/mL (equivalent to 0.01% sample content) for each of the 130 analytes and corresponded to the lowest validated concentration level. Only two analytes—5F-NPB-22 and FUB-NPB-22—failed to meet the LOQ criterion (signal-to-noise ratio of 10:1) for a fortified concentration level of 0.1 ng/mL.

#### 2.3.2. Calibration Model

**Figure 4:**
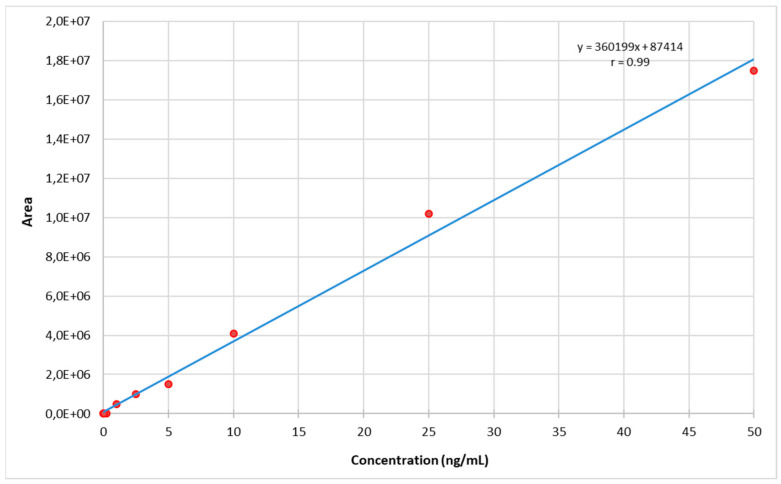
Calibration curve for THC.

The calibration curves of all cannabinoids were constructed by plotting the peak areas corresponding with the analyte concentrations based on three independent solution injections for each calibration point: 10-point calibration curves for natural cannabinoids over the 0.05–50 ng/mL range, 7-point calibration curves for 117 synthetic cannabinoids over the 0.05–0.5 ng/mL range, and 7-point calibration curves for 2 synthetic cannabinoids (i.e., 5F-NPB-22 and FUB-NPB-22) over the 0.5–50 ng/mL range were fitted with a quadratic equation (weight × 1/concentration) to minimalize the calculation error at low concentrations, with accuracy of 80–120% for each calibration point. All of the calibration curves showed correlation coefficients higher than 0.99 in the working range (Figure 4, Table S5).

#### 2.3.3. Precision and Bias

Bias and precision (%CV) studies were carried out concurrently. Measurements were performed for six samples, with four spiking levels for natural cannabinoids and three for synthetic cannabinoids (low, medium, and high). A limit of 20% for %CV was used, while the maximum acceptable bias was ±20%. Expanded uncertainty (U) was calculated based on the uncertainty component for the bias and precision and reported at k = 2, providing an approximate 95% confidence level. A summary of the obtained results for analytes that met the validation criteria is presented in Table S4. All 13 natural cannabinoids met the validation criteria. Values of bias ranged from −11.67% to 13.00% at low concentration levels (0.1 ng/mL, 0.01%), from −19.40% to −7.10% and from −19.48% to −13.94% at medium concentration levels (0.5 ng/mL, 0.05% and 5 ng/mL, 0.5%, respectively), and from −19.37% to −7.93% at high concentration levels (50 ng/mL, 5%), while the maximum %CV was 15.19%. Out of 119 synthetic cannabinoids, only 5F-NPB-22 and FUB-NPB-22 did not meet the criteria for the bias and precision. The values of bias for 117 synthetic cannabinoids ranged from −14.00% to 17.00% at low concentration levels (0.1 ng/mL, 0.01%), from −15.60% to 19.20% at medium concentration levels (0.5 ng/mL, 0.05%), and from −16.04% to −5.68% at high concentration levels (5 ng/mL, 0.5%), with %CV from 1.79% to 19.77%. Expanded uncertainty did not exceed 40% for 130 compounds.

#### 2.3.4. Carryover

Blank samples were injected directly after the sample with the highest level of analytes to determinate any carryover effects. No peaks of 130 target compounds at their respective retention times for each analyte were found. No carryover was observed in the developed method.

#### 2.3.5. Matrix Effect

Standard calibration curves and matrix-matched calibration curves were compared. Suppression or enhancement of ionization for each analyte was studied. Each point of the matrix calibration curves was prepared by 100,000-fold dilution with blank oil, and the same with the real oil samples. There were no statistically significant differences between these two types of curve for any of the analytes. Therefore, calibration curves in the solvent were selected and ultimately accepted in the validation process.

#### 2.3.6. Dilution Integrity

Dilution integrity was evaluated in the event that the studied sample concentrations were above the upper limit of quantification for basic sample dilution. Six samples at four spiking levels for 1,000,000-fold dilution were tested for 13 natural cannabinoids. The obtained results met the criteria for the bias and %CV, and the measurement uncertainty did not exceed 40% for any of the analytes. Therefore, this dilution can be use in routine analyses to ensure the determination of high concentrations of naturally occurring cannabinoids in cannabis products in a range from 0.1% to 50% (Tables S4 and S5).

### 2.4. Application to Dietary Supplements and Food Samples

Cannabis products in oil form were tested with the developed method. OF 12 samples, 8 were oils (Samples 1, 2, 3, 4, 5, 6, 11, and 12) and 4 were oil-filled capsules (Samples 7, 8, 9, and 10). Five products were sold as dietary supplements (Samples 2, 7, 8, 9, and 10). For each tested product, two independent samples for both dilutions were analyzed. The differences in quantitative results for duplicate samples did not exceed 20%. The results obtained for Samples 1, 2, 3, 4, 5, 7, 8, 9, and 12 (±expanded uncertainty) for total CBD content (i.e., CBD or sum of CBD and CBDA) were consistent with those declared on the label (Samples 1, 2, 3, 4, 5, and 12: 30%, 5%, 15%, 10%, 5%, and 10%, respectively; Samples 7, 8, and 9: 1.5%, 2%, and 1%, respectively—calculated from 10 mg per capsule to % content in oil). THC or THC/THCA-A was found in eight samples (2, 3, 4, 5, 6, 8, 9, and 12), despite there being no indication on these products’ labels that they may contain THC. The total contents of CBD and CBG for Sample 6 matched those declared by the manufacturer. Sample 11 was a hemp-seed-based cooking oil; no cannabinoids were detected at concentrations above 0.01%—the same as in Sample 10 (hemp seed oil capsules). None of the 117 synthetic cannabinoids were detected above the limit of quantification (0.01%) in any of the samples (Table 1).

The dynamic development of mass spectrometry has made it the leading analytical technique used in the study of food adulteration—including dietary supplements. However, due to the ever-increasing number of illegal additives in the form of psychoactive substances and drugs, the trends in the analysis of these compounds have changed in recent years. The use of gas chromatography coupled with mass spectrometry (GC–MS) is often limited to screening tests that require additional confirmatory analysis, which significantly extends the research time. On the other hand, quantitative analysis by GC–MS—as in the case of liquid chromatography coupled with mass spectrometry (LC–MS)—usually involves one or several compounds during one chromatographic run, which is a drop in the ocean relative to current analytical needs. Moreover, the sensitivity of this apparatus is often insufficient, and the preparation of samples is complicated and time-consuming. The solution to a large part of these problems is the combination of liquid chromatography techniques with tandem mass spectrometry (LC–MS/MS). The use of the MRM mode enables the qualitative and quantitative analysis of many compounds during one chromatographic run.

Current research on cannabis products focuses mainly on determining the contents of cannabinoids that occur naturally in cannabis, usually covering up to a dozen compounds at a time [27,34,36,37,38,46,47]. Heo et al. [33] published a method used for the determination of ∆9-tetrahydrocannabinol (THC) and eight synthetic cannabinoids, indicating that compounds of this type were detected by the Korean Customs Service in herbal products and dietary supplements in 2012. Despite numerous scientific reports on methods of detecting synthetic cannabinoids in biological materials such as saliva, blood, urine, or hair, there is not much information on such methods being researched or implemented in the field of dietary supplements or food products made of hemp.

That being the case, by utilizing the LC–MS/MS technique, we developed an innovative method for the detection and quantification of 13 natural cannabinoids and 117 synthetic cannabinoids in dietary supplements and food products in the form of oils during one 13-min chromatographic run. For all 130 compounds, the limit of quantification was determined at the level of 100 µg/g, which corresponds to a 0.01% *w*/*w* of the tested oils. Taking into the account the nature of the tested compounds, two working ranges of the calibration curves were established. Due to their expected presence in cannabis products, the widest possible working range of the calibration curve was determined for natural cannabinoids: 0.1–50 ng/mL (0.01–5%) for the stock dilution. Considering the high concentrations of some cannabinoids in CBD oils—especially cannabidiol—an additional dilution was validated to extend the quantitative analysis to 50%. In the case of synthetic cannabinoids, due to the fact that their use in dietary supplements is not allowed, the focus was on lower levels. Hence, a shorter working range of the calibration curves was used for these compounds: 0.1–5 ng/mL, which corresponds to 0.01–5% *w*/*w* of the tested oils.

Ten hemp products were analyzed with the newly developed method; in the case of seven of them—including five in the form of oils and two in the form of capsules containing oil—the presence of the psychoactive ∆9-tetrahydrocannabinol (THC) was detected at concentrations ranging from 0.02% to 0.09%. The obtained results confirm doubts regarding the quality and safety of these hemp-derived products. In recent years, the GIS (the main sanitary inspectorate of Poland) has repeatedly withdrawn these types of products from the market, including oils containing prohibited hemp extract from *Cannabis sativa* var. sativa, in which the content of Δ9-THC was found at levels of 0.9%, 0.12%, 0.15%, or 0.16%.

## 3. Materials and Methods

Based on a scientific literature review and experience gained at the Institute of Forensic Genetics in Bydgoszcz (Poland) with research in the fields of forensic physicochemistry and toxicology, 130 chemical compounds were selected for this study, including psychoactive substances, active substances of drugs, and cannabinoids that occur naturally in cannabis. The type and range of compounds constituting the basis of the analytical method were selected depending on the analyzed matrix. In addition, 8 chemical compounds belonging to the pesticide group were selected and used to control the retention times of the developed analytical procedure (i.e., internal controls). Certified reference standards of all of the tested analytes (purity ≥ 98%) were obtained from the following companies: LGC GmbH (Luckenwalde, Germany), Lipomed AG (Arlesheim, Switzerland), Cayman Chemical (Ann Arbor, MI, USA), Chiron AS (Trondheim, Norway), Dr. Ehrenstorfer (Augsburg, Germany), Cerilliant (Round Rock, TX, USA), and TRC (North York, Toronto, ON, Canada). Ultrapure water was prepared using a Milli-Q Simplicity^®^ purification system (Millipore, Billerica, MA, USA). HPLC-grade methanol and acetonitrile were purchased from Chemsolve (Łódź, Poland). LC–MS eluent additives—formic acid and ammonium formate—were obtained from CHEM LAB NV (Zedelgem, Belgium). Twelve hemp-containing supplements in the form of oils and capsules containing oil were purchased online and stored before testing according to the manufacturer’s recommendations (i.e., dark and dry place, room temperature).

### 3.1. Preparation of Standards and Solutions

Individual standard stock solutions were prepared by dissolving each compound in methanol at a concentration of 1 mg/mL. Working standard solutions were prepared by combining all of the stock solutions and diluting the mixture in methanol to a concentration appropriate for the preparation of calibration curves and method validation. All of the standard solutions were stored at ≤−20 °C. Atrazine was used as an internal standard.

### 3.2. Sample Preparation

Portions of oil samples (100 ± 0.5 mg) were weighed into 2 mL Eppendorf tubes. Afterwards, 1 mL of acetonitrile with the internal standard was added, vortexed, and shaken using an Eppendorf ThermoMixer for 10 min (21 °C, 1400 RPM). To provide a good phase separation, the samples were stored at −20 °C for 10 min. After freezing, the acetonitrile layer was quickly diluted 100,000-fold and 1,000,000-fold (final dilution mixed with the mobile phase).

### 3.3. HPLC

High-performance liquid chromatography (HPLC) was carried out using a Sciex ExionLC^TM^ (Framingham, MA, USA) system equipped with two binary pumps, a solvent degasser, an autosampler with temperature control, a controller, and a column heater. Chromatographic separation of all of the analytes was performed using a Kinetex C18 column (100 × 3 mm, 2.6 µm particle size). The column temperature was maintained at 35 °C, and the injection volume was 20 µL. The autosampler temperature was set to 4 °C. The mobile phases were water (phase A) and methanol (phase B); both contained 0.1% formic acid and 2 mM ammonium formate. The LC pump was programmed at a flow rate of 0.5 mL/min, and the initial composition was 40% A and 60% B. A gradient elution was performed, where phase B was increased linearly to 90% in the first 7 min, and then it was maintained for 2 min. Finally, the gradient was returned to the initial composition, and the LC system was stabilized for 3.5 min before the next injection. The total run time was 13 min. A stock standard solution of each analyte was analyzed individually to determine its unique retention time.

### 3.4. MS/MS

Detection of all of the analytes with superior quantitative results in a single injection workflow was accomplished using a SCIEX Triple Quad^TM^ 5500+ triple-quadrupole mass spectrometer (Framingham, MA, USA) operated in multiple reaction monitoring (MRM) mode. A Turbo V™ source was used with an electrospray ionization (ESI) probe in positive polarity, and the parameters were optimized for optimal sensitivity. The ion source temperature and the ion spray voltage were set to 550 °C and 4500 V, respectively. Ion source gases 1 and 2 were both set to 50 psi. Nitrogen was used as the collision gas and the curtain gas, with a flow ratio of 9 psi and 30 psi, respectively. All other detection settings—such as molecular ions (Q1 mass) (*m*/*z*), product ions (Q3 mass) (*m*/*z*), declustering potential (DP) (V), entrance potential (EP) (V), collision energy (CE) (V) and collision cell exit potential (CXP) (V)—were optimized individually for 130 cannabinoids by infusing the standard solutions into the mobile phase, which was directed to the ESI source via an infusion pump. Analyst^®^ software version 1.7.2 was used for data acquisition. LC–MS/MS data were processed using the MultiQuant™ software version 3.0.3.

### 3.5. Validation

The developed method was validated according to the recommendations of the Scientific Working Group of Forensic Toxicology (SWGTOX) [48]. The selectivity, specificity, calibration model, limit of quantification, precision, BIAS, carryover, matrix effect, stability, and dilution integrity were assessed. Validation was performed using rapeseed oil as a blank matrix.

## 4. Conclusions

The application of advanced analytical equipment, such as the LC–MS/MS system, made it possible for us to develop an innovative method of detecting and quantifying as many as 130 cannabinoids (13 natural and 117 synthetic) in dietary supplements in the form of oils, during a single 13-min chromatographic run. In addition, the universal extraction method enabled the simultaneous isolation of all 130 analytes belonging to different groups of chemical compounds from the matrix, significantly reducing the sample preparation time required for research. The developed method was characterized not only by the ability to test a wide range of compounds in a relatively short time, but also by high sensitivity. This allows for the quantification of each of the 130 analyzed compounds at the level of 100 µg/g of the tested dietary supplement in the form of oils, i.e., 0.01% of their mass. Establishing the same working range of the calibration curves (0.05–50 ng/mL) for all of the tested compounds and meeting the validation requirements, significantly improved the work both during the preparation of the test samples and during the analysis of the obtained data. The developed procedure was successfully applied in the analysis of 12 cannabis products. This method will be very useful for monitoring the dietary supplement market.

## Figures and Tables

**Table 1:** Analysis of cannabinoids concentration in cannabis oil samples.

Compound	Cannabis Oil Samples											
	1	2	3	4	5	6	7	8	9	10	11	12
DELTA-9-TETRAHYDROCANNABINOL (THC)	<0.01	0.03	0.07	0.07	0.09	0.09	<0.01	0.02	0.02	<0.01	<0.01	0.06
DELTA-9-TETRAHYDROCANNABINOL ACID (THCA-A)	<0.01	<0.01	<0.01	<0.01	<0.01	<0.01	<0.01	<0.01	<0.01	<0.01	<0.01	0.01
CANNABIDIOL (CBD)	28.38	3.85	15.71	10.75	7.32	4.74	1.54	2.04	0.84	<0.01	<0.01	6.09
CANNABIDIOLIC ACID (CBDA)	0.01	1.63	0.26	<0.01	0.66	0.01	<0.01	<0.01	0.33	<0.01	<0.01	2.41
CANNABIGEROL (CBG)	0.02	0.01	<0.01	0.32	1.12	5.06	0.16	0.05	0.01	<0.01	<0.01	0.23
CANNABIGEROLIC ACID (CBGA)	<0.01	<0.01	<0.01	<0.01	0.06	<0.01	<0.01	<0.01	<0.01	<0.01	<0.01	0.02
CANNABICHROMENE (CBC)	<0.01	0.05	0.13	0.12	0.19	0.21	<0.01	0.08	0.03	<0.01	<0.01	0.11
CANNABIVARIN (CBV)	<0.01	<0.01	<0.01	<0.01	<0.01	<0.01	<0.01	<0.01	<0.01	<0.01	<0.01	<0.01
CANNABIDIVARINIC ACID (CBDVA)	<0.01	0.02	<0.01	<0.01	<0.01	<0.01	<0.01	<0.01	<0.01	<0.01	<0.01	0.03
CANNABIGEROVARINIC ACID (CBGVA)	<0.01	<0.01	<0.01	<0.01	<0.01	<0.01	<0.01	<0.01	<0.01	<0.01	<0.01	<0.01
CANNABINOL (CBN)	<0.01	<0.01	<0.01	<0.01	<0.01	0.04	<0.01	<0.01	<0.01	<0.01	<0.01	<0.01
TETRAHYDROCANNABIVARINIC ACID (THCVA)	<0.01	<0.01	<0.01	<0.01	<0.01	<0.01	<0.01	<0.01	<0.01	<0.01	<0.01	<0.01
TETRAHYDROCANNABIVARIN (THCV)	<0.01	<0.01	<0.01	<0.01	<0.01	<0.01	<0.01	<0.01	<0.01	<0.01	<0.01	<0.01
SYNTHETIC CANNABINOIDS (117 COMPOUNDS)	<0.01	<0.01	<0.01	<0.01	<0.01	<0.01	<0.01	<0.01	<0.01	<0.01	<0.01	<0.01

## Data Availability

The data presented in this study are available in supplementary material.

## Supplementary Materials

The following supporting information can be downloaded at: https://www.mdpi.com/article/10.3390/molecules27238601/s1, Table S1: LC–MS/MS method parameters for 13 natural and 119 synthetic cannabinoids; Table S2: The volume of the extractant and extraction recovery; Table S3: The extraction time and compounds of recovery; Table S4: Summary of the validation results; Table S5: Summary of the validation results of dilution integrity.

## Sample Availability

Samples of the compounds are available from the authors.
