# Supplementary material for: Development and Validation of the LC–MS/MS Method for Determination of 130 Natural and Synthetic Cannabinoids in Cannabis Oil
Source: Molecules. 2022 Dec 6;27(23):8601. doi: 10.3390/molecules27238601 (PMC9736437; doi:10.3390/molecules27238601)
Supplement: Supplementary file 1 [file molecules-27-08601-s001.zip › molecules-2001717-supplementary.pdf]

Supplementary materials

Table S1. LC-MS/MS method parameters for 13 natural and 119 synthetic cannabinoids

COMPOUND	RT (min.)	Q1 MASS (M/Z)	Q3 MASS (M/Z)	DP (V)	EP (V)	CE (V)	CXP (V)
DELTA-9-TETRAHYDROCANNABINOL (THC)	8.73	315.2	193.2	106	10	33	12
			123.1	106	10	45	14
DELTA-9-TETRAHYDROCANNABINOLIC ACID (THCA-A)	10.31	35.2	219.2	91	10	43	14
			341.2	91	10	21	12
CANNABIDIOL (CBD)	7.43	315.2	193.2	100	10	29	12
			123.1	100	10	43	6
CANNABIDIOLIC ACID (CBDA)	7.72	359.2	219.2	81	10	41	14
			341.2	81	10	21	22
CANNABIGEROL (CBG)	7.50	317.2	193.2	91	10	23	12
			123.1	91	10	43	14
CANNABIGEROLIC ACID (CBGA)	8.17	361.2	219.1	76	10	37	14
			343.2	76	10	17	24
CANNABICHROMENE (CBC)	9.29	315.2	193.2	61	10	29	12
			259.2	61	10	21	18
CANNABIVARIN (CBV)	6.90	283.2	241.2	101	10	25	16
			223.2	101	10	29	14
CANNABIDIVARINIC ACID (CBDVA)	6.74	331.1	191.1	81	10	41	12
			313.2	81	10	19	18
CANNABIGEROVARINIC ACID (CBGVA)	7.34	333.1	191.1	76	10	37	12
			315.2	76	10	15	18
CANNABINOL (CBN)	8.34	311.2	223.2	106	10	29	14
			293.2	106	10	25	18
TETRAHYDROCANNABIVARINIC ACID (THCVA)	8.87	331.1	191.2	71	10	43	12
			313.2	71	10	21	20
TETRAHYDROCANNABIVARIN (THCV)	7.56	287.2	165.2	86	10	31	16
			123.1	86	10	43	6
3-CAF	7.64	383.0	239.0	95	10	25	20
			210.1	95	10	65	12
5-CHLORO AB-PINACA	4.49	365.1	320.1	95	10	23	22
			249.1	95	10	35	22

5-CHLORO-NNEI	6.01	391.1	248.1	45	10	31	20
			144.0	45	10	57	16
5F-ABICA	3.41	348.1	232.0	10	10	29	20
			144.1	71	10	53	18
5F-AB-PINACA	3.53	349.0	304.1	75	10	23	22
			233.1	75	10	33	18
5F-ADB (5F-MDMB-PINACA)	5.87	377.9	233.1	110	10	35	16
			318.2	110	10	25	22
5F-AMB (5F-AMB-PINACA)	5.14	364.1	233.1	105	10	31	20
			304.1	105	10	23	28
5F-APICA (STS-135)	7.29	383.1	135.1	140	10	41	16
			232.1	140	10	35	20
5F-APINACA (AKB-48-5F)	7.91	384.1	135.1	30	10	31	14
			93.1	30	10	29	10
5-F-JWH-018 ADAMANTYL ANALOG	7.68	368.1	135.1	135	10	41	14
			93.1	135	10	61	10
5-FLUORO MN-24 (5-FLUORO NNEI)	5.24	375.1	232.1	30	10	33	20
			144.1	30	10	55	14
5-FLUORO THJ	7.36	377.3	233.1	91	10	29	12
			145.1	91	10	53	14
5-FLUORO-2-ADB-PINACA_ISOMER 2	3.62	363.2	145.0	110	10	47	16
			318.1	110	10	29	22
5-FLUORO-CUMYL-PICA	5.56	367.1	249.1	100	10	23	14
			206.2	100	10	37	16
5-FLUORO-MN-18	6.65	376.1	233.1	95	10	25	18
			145.0	95	10	53	16
5-FLUORO-MN-21 (5-FLUORO-PCN)	1.38	376.1	145.1	155	10	55	14
			356.0	155	10	45	28
5-FLUORO-SDB-006	4.56	339.0	232.1	125	10	31	16
			91.1	125	10	55	12
5F-NPB-22	4.82	378.1	233.1	35	10	27	20
			213.2	35	10	39	18
5F-PB-22	5.28	377.1	232.1	81	10	27	10
			144.1	81	10	57	20
5-F-PENTYL-3-PYRIDINOYLINDOLE	3.41	311.0	144..1	140	10	53	16

			232.1	140	10	43	20
5F-SDB-005	6.95	377.1	233.1	100	10	21	18
			145.1	100	10	51	16
A-796260	3.02	355.0	125.1	155	10	29	16
			114.2	155	10	37	14
A-834735	6.11	340.3	125.1	156	10	29	16
			55.0	156	10	61	10
A-836339	3.89	311.0	187.1	10	10	27	18
			125.1	10	10	35	14
AB-001	8.90	350.1	135.2	10	10	39	16
			79.1	10	10	63	10
AB005	2.59	353.1	112.1	125	10	31	12
			98.1	125	10	47	12
AB-CHMINACA	5.98	357.1	241.2	30	10	35	18
			312.2	30	10	23	20
AB-FUBINACA	4.06	369.1	324.1	160	10	23	22
			253.0	160	10	33	20
AB-FUBINACA 2-FLUOROBENZYL ISOMER	4.30	369.2	253.0	86	10	33	12
			324.2	86	10	19	14
AB-FUBINACA 3-FLUOROBENZYL ISOMER	4.05	369.1	253.0	95	10	35	22
			324.1	95	10	23	22
AB-PINACA	5.14	331.1	215.1	85	10	33	18
			286.2	85	10	21	22
ADB-FUBINACA	4.86	383.2	338.1	75	10	23	22
			253.1	75	10	35	20
ADB-PINACA	5.81	345.1	145.1	40	10	50	22
			215.1	90	10	35	14
ADB-PINACA ISOMER 1	5.41	345.1	215.1	90	10	35	14
			300.2	71	10	19	14
ADB-PINACA ISOMER 2	5.81	345.1	215.1	90	10	35	14
			300.2	71	10	19	14
ADB-PINACA ISOMER 3	5.86	345.1	215.1	90	10	35	14
			300.2	71	10	19	14
ADB-PINACA ISOMER 4	5.90	345.1	215.1	90	10	35	14
			145.1	40	10	50	22

AKB48_N-(4-FLUOROBENZYL) ANALOG	8.20	404.2	135.2	106	10	27	14
			93.1	106	10	69	12
AM1220	1.68	383.1	98.1	130	10	45	12
			112.1	130	10	29	12
AM-1248	3.35	391.1	135.1	40	10	39	14
			112.1	40	10	39	14
AM-2201	6.04	359.9	155.0	145	10	35	16
			127.1	145	10	61	16
AM2201 8-QUINOLINYL CARBOXAMIDE	7.10	376.1	232.0	50	10	27	16
			144.0	50	10	55	16
AM2201 BENZIMIDAZOLE ANALOG	6.42	361.1	155.0	10	10	41	16
			127.1	10	10	67	14
AM2232	4.13	353.0	155.0	135	10	33	16
			127.0	135	10	61	14
BB-22	7.39	385.1	240.1	10	10	25	14
			144.0	10	10	51	16
CB-13	9.75	368.9	155.0	140	10	35	18
			171.0	140	10	37	18
CBL-018	8.47	358.1	214.1	95	10	27	18
			144.1	95	10	51	16
CUMYL-PICA	6.84	349.2	231.2	100	10	23	18
			188.2	100	10	35	16
EAM-2201	7.17	388.1	183.1	145	10	37	18
			232.1	145	10	37	20
EG-2201	8.22	410.1	155.1	35	10	35	14
			127.1	35	10	65	14
FDU-PB-22	7.60	396.2	252.1	10	10	19	24
			109.1	10	10	43	16
FUB-144	7.22	350.1	109.1	140	10	47	12
			125.1	140	10	31	14
FUB-JWH-018	6.50	380.0	155.0	130	10	33	16
			109.1	130	10	51	12
FUB-NPB-22	5.34	398.1	253.0	10	10	25	18
			109.0	10	10	45	12
FUB-PB-22	5.76	397.1	252.1	25	10	21	16

			109.1	25	10	45	14
HU-210	8.71	387.2	243.1	146	10	25	12
			43.0	146	10	71	8
JWH 018 BENZIMIDAZOLE ANALOG	7.86	343.1	155.0	155	10	41	16
			127.1	155	10	67	14
JWH-011	8.44	384.1	155.1	125	10	37	18
			127.1	125	10	67	14
JWH-016	7.10	342.1	155.0	130	10	35	18
			127.1	130	10	61	14
JWH-018	7.46	342.1	155.0	125	10	35	14
			127.1	125	10	59	14
JWH-018 ADAMANTYL CARBOXAMIDE	8.34	365.2	135.1	160	10	39	14
			214.1	160	10	33	20
JWH-020	8.59	370.2	155.1	110	10	19	26
			127.1	110	10	29	20
JWH-022	6.87	340.1	155.0	150	10	33	16
			127.1	150	10	59	14
JWH-031	7.02	306.1	155.0	110	10	29	16
			127.0	110	10	57	16
JWH-071	5.27	300.1	155.1	10	10	31	10
			127.0	10	10	53	14
JWH-073	6.80	328.1	127.1	131	10	63	16
			155.1	131	10	31	20
JWH-080	7.16	358.1	185.1	20	10	39	18
			157.2	20	10	51	18
JWH-081	7.77	372.1	185.1	110	10	37	16
			214.1	110	10	35	18
JWH-098	7.98	385.9	185.2	125	10	35	12
			157.1	125	10	57	18
JWH-116	8.23	370.0	155.1	140	10	35	18
			127.1	140	10	65	16
JWH-122	8.01	356.2	169.1	150	10	35	16
			141.0	150	10	57	14
JWH-145	8.12	368.1	155.1	125	10	29	14
			127.1	125	10	63	16

JWH-146	9.14	396.1	155.1	155	10	31	14
			127.1	155	10	65	14
JWH-147	8.61	382.2	155.0	136	10	27	20
			127.1	136	10	69	16
JWH-149	8.21	370.1	169.1	140	10	37	16
			141.1	140	10	57	16
JWH-167	6.60	306.1	91.1	130	10	33	10
			214.1	130	10	35	12
JWH-175	10.51	328.3	141.1	106	10	29	18
			115.1	106	10	83	14
JWH-182	8.87	384.1	197.1	150	10	37	12
			141.1	150	10	59	14
JWH-193	3.05	399.1	169.1	10	10	33	16
			114.2	10	10	35	10
JWH-198	2.80	415.1	185.1	181	10	33	22
			114.2	181	10	35	10
JWH-200	2.35	385.0	155.0	110	10	31	16
			114.1	110	10	35	14
JWH-201	6.47	336.1	121.1	145	10	35	14
			135.0	145	10	37	16
JWH-213	8.62	384.1	183.1	140	10	37	16
			228.2	140	10	37	20
JWH-307	7.95	386.0	155.0	115	10	29	18
			127.0	115	10	63	16
JWH-309	8.92	418.1	155.1	150	10	31	16
			127.1	150	10	67	14
JWH-368	8.15	386.1	155.0	95	10	29	16
			127.1	95	10	65	14
JWH-369	8.28	402.1	155.0	115	10	31	16
			127.1	115	10	67	14
JWH-412	7.86	360.1	173.0	140	10	37	18
			145.0	140	10	63	16
JWH-424	7.17	422.0	235.0	140	10	37	20
			233.0	160	10	39	16
MAM-2201	6.66	374.1	169.1	40	10	37	16

			141.0	40	10	59	18
MDMB-CHMICA (MMB-CHMINACA)	7.48	385.1	240.1	95	10	25	16
			144.0	95	10	55	18
MDMB-CHMINACA	7.96	386.1	241.1	115	10	37	20
			326.2	115	10	25	6
MEPIRAPIM	1.61	314.1	214.2	20	10	25	18
			144.1	20	10	47	18
MMB018	6.06	345.1	214.1	10	10	23	18
			144.1	10	10	49	18
MMB2201	4.63	363.1	232.1	10	10	23	14
			144.1	10	10	53	18
MN-18	8.01	358.0	215.1	110	10	27	18
			145.1	110	10	47	16
MN-25	4.20	440.2	261.1	155	10	35	16
			114.1	155	10	43	14
MN-25-2-METHYL DERIVATIVE	4.75	454.2	275.1	170	10	33	18
			114.1	170	10	41	12
MO-CHMINACA	7.95	387.1	241.1	115	10	27	20
			145.1	115	10	49	18
NM2201	7.27	376.1	232.1	90	10	21	20
			144.1	90	10	53	16
NNEI	6.53	357.1	214.2	125	10	31	16
			144.1	125	10	53	16
ORG-28611	2.71	384.2	270.1	10	10	27	22
			174.1	10	10	49	18
PB-22	6.67	359.0	214.1	10	10	21	14
			144.0	10	10	51	16
PSB-SB-1202	8.65	367.1	259.0	100	10	29	18
			121.1	100	10	23	14
PTI-1	3.87	356.1	283.1	110	10	31	20
			213.2	110	10	49	14
PTI-2	4.19	400.1	283.1	25	10	33	20
			213.1	25	10	55	16
PX-1	4.02	396.1	232.1	35	10	31	18
			379.2	35	10	15	12

PX-2	4.16	397.1	233.1	10	10	35	18
			352.1	10	10	23	24
RCS-4	6.67	322.1	135.1	25	10	33	14
			77.1	25	10	63	12
SDB-005	8.08	359.1	215.1	100	10	23	18
			145.0	100	10	47	16
SDB-006	5.98	321.1	91.0	130	10	49	12
			214.1	130	10	29	20
THJ	8.61	359.1	215.1	115	10	31	16
			145.0	115	10	49	16
THJ-018	8.01	343.1	215.1	110	10	27	18
			145.1	110	10	43	14
THJ-2201 (5-FLUORO THJ-018)	6.69	361.1	233.1	25	10	25	22
			145.0	25	10	49	18
UR-144	8.09	312.1	125.1	115	10	31	14
			214.1	115	10	33	18
WIN 54.461	3.75	459.0	135.1	20	10	29	14
			114.0	20	10	45	14
WIN 55.212-2	5.34	427.1	155.1	145	10	33	16
			127.1	145	10	69	14
XLR-11	6.84	330.1	125.1	85	10	33	14
			232.0	85	10	35	14
XLR12	7.01	352.1	125.2	120	10	33	14
			254.0	120	10	37	22
ATRAZINE	2.71	216.1	174.0	126	10	23	24

Table S2. The volume of the extractant and extraction recovery.

No.	COMPOUND	VOLUME OF EXTRACTANT [mL] 1	5	10
		RECOVERY [%]		
**1.**	Δ-9-TETRAHYDROCANNABINOL (THC)	112.67	86.70	81.18
**2.**	Δ-9-TETRAHYDROCANNABINOL ACID (THCA-A)	88.33	85.17	80.52

No.	COMPOUND	VOLUME OF EXTRACTANT [mL]		
		1	5	10
		RECOVERY [%]		
**3.**	CANNABIDIOL (CBD)	102.67	92.90	84.96
**4.**	CANNABIDIOLIC ACID (CBDA)	91.17	80.60	81.01
**5.**	CANNABIGEROL (CBG)	95.50	89.47	84.60
**6.**	CANNABIGEROLIC ACID (CBGA)	113.00	90.03	81.91
**7.**	CANNABICHROMENE (CBC)	103.83	87.17	81.94
**8.**	CANNABIVARIN (CBV)	93.83	91.60	86.06
**9.**	CANNABIDIVARINIC ACID (CBDVA)	102.83	89.87	82.22
**10.**	CANNABIGEROVARINIC ACID (CBGVA)	100.17	89.20	81.63
**11.**	CANNABINOL (CBN)	93.17	92.10	85.68
**12.**	TETRAHYDROCANNABIVARINIC ACID (THCVA)	99.17	88.90	83.14
**13.**	TETRAHYDROCANNABIVARIN (THCV)	99.67	89.37	83.17
**14.**	3-CAF	88.33	91.33	93.07
**15.**	5-CHLORO AB-PINACA	91.83	92.60	94.67
**16.**	5-CHLORO-NNEI	92.17	91.07	91.89
**17.**	5F-ABICA	93.17	92.50	92.19
**18.**	5F-AB-PINACA	94.17	91.50	92.15
**19.**	5F-ADB (5F-MDMB-PINACA)	92.17	89.67	90.71
**20.**	5F-AMB (5F-AMB-PINACA)	92.50	90.87	89.11
**21.**	5F-APICA (STS-135)	91.17	90.77	88.72
**22.**	5F-APINACA (AKB-48-5F)	91.33	90.50	88.07
**23.**	5-F-JWH-018 ADAMANTYL ANALOG	90.83	85.80	88.85
**24.**	5-FLUORO MN-24 (5-FLUORO NNEI)	92.33	90.37	92.65
**25.**	5-FLUORO THJ	103.33	91.20	88.20
**26.**	5-FLUORO-2-ADB-PINACA ISOMER 2	92.00	90.60	93.60
**27.**	5-FLUORO-CUMYL-PICA	92.67	88.13	91.64

No.	COMPOUND	VOLUME OF EXTRACTANT [mL]		
		1	5	10
		RECOVERY [%]		
**28.**	5-FLUORO-MN-18	92.83	92.03	91.18
**29.**	5-FLUORO-MN-21 (5-FLUORO-PCN)	104.33	101.97	94.97
**30.**	5-FLUORO-SDB-006	93.83	91.87	89.48
**31.**	5F-PB-22	93.17	90.70	92.96
**32.**	5-F-PENTYL-3-PYRIDINOYLINDOLE	94.00	92.67	92.89
**33.**	5F-SDB-005	95.83	92.93	93.11
**34.**	A-796260	97.83	93.47	93.98
**35.**	A-834735	91.33	88.97	91.91
**36.**	A-836339	93.67	88.93	91.31
**37.**	AB-001	92.67	89.93	91.62
**38.**	AB005	115.33	105.87	100.75
**39.**	AB-CHMINACA	92.50	91.43	91.32
**40.**	AB-FUBINACA	86.00	94.07	94.61
**41.**	AB-FUBINACA 2-FLUOROBENZYL ISOMER	93.67	92.10	94.22
**42.**	AB-FUBINACA 3-FLUOROBENZYL ISOMER	92.83	93.27	93.28
**43.**	AB-PINACA	94.00	91.67	89.43
**44.**	ADB-FUBINACA	93.83	90.87	91.74
**45.**	ADB-PINACA	92.67	90.03	90.47
**46.**	ADB-PINACA ISOMER 1	92.33	90.73	90.50
**47.**	ADB-PINACA ISOMER 2	92.50	91.23	91.30
**48.**	ADB-PINACA ISOMER 3	94.17	89.73	92.01
**49.**	ADB-PINACA ISOMER 4	93.33	89.77	90.59
**50.**	AKB48_N-(4-FLUOROBENZYL) ANALOG	89.83	86.27	85.02
**51.**	AM1220	115.67	111.57	101.63
**52.**	AM-1248	112.67	104.37	98.59

No.	COMPOUND	VOLUME OF EXTRACTANT [mL]		
		1	5	10
		RECOVERY [%]		
**53.**	AM-2201	92.50	90.77	91.19
**54.**	AM2201 8-QUINOLINYL CARBOXAMIDE	93.33	93.83	93.17
**55.**	AM2201 BENZIMIDAZOLE ANALOG	91.17	90.83	91.15
**56.**	AM2232	92.33	92.13	90.78
**57.**	BB-22	91.33	88.10	91.54
**58.**	CB-13	89.33	88.10	86.88
**59.**	CBL-018	91.50	90.63	89.82
**60.**	CUMYL-PICA	93.33	90.83	90.81
**61.**	EAM-2201	92.33	90.87	88.90
**62.**	EG-2201	87.00	88.13	83.96
**63.**	FDU-PB-22	86.33	88.60	91.01
**64.**	FUB-144	90.00	89.30	90.37
**65.**	FUB-JWH-018	92.17	91.07	89.47
**66.**	FUB-PB-22	95.17	91.80	90.32
**67.**	HU-210	90.50	84.77	89.97
**68.**	JWH 018 BENZIMIDAZOLE ANALOG	91.50	90.67	90.23
**69.**	JWH-011	92.17	90.30	91.00
**70.**	JWH-016	92.50	88.53	90.03
**71.**	JWH-018	92.50	87.67	89.18
**72.**	JWH-018 ADAMANTYL CARBOXAMIDE	95.67	93.97	93.34
**73.**	JWH-020	95.67	90.50	89.45
**74.**	JWH-022	96.33	91.33	91.78
**75.**	JWH-031	92.50	90.00	91.00
**76.**	JWH-071	93.67	90.70	90.13
**77.**	JWH-073	91.67	90.97	90.94

No.	COMPOUND	VOLUME OF EXTRACTANT [mL]		
		1	5	10
		RECOVERY [%]		
**78.**	JWH-080	93.17	90.80	91.77
**79.**	JWH-081	92.33	90.83	89.28
**80.**	JWH-098	92.50	91.00	87.66
**81.**	JWH-116	91.67	89.17	88.76
**82.**	JWH-122	91.33	88.83	86.77
**83.**	JWH-145	90.33	88.93	88.07
**84.**	JWH-146	91.50	87.40	88.34
**85.**	JWH-147	91.67	88.80	86.20
**86.**	JWH-149	89.17	86.53	91.61
**87.**	JWH-167	93.67	90.93	90.84
**88.**	JWH-175	103.50	100.30	88.51
**89.**	JWH-182	92.83	92.70	92.38
**90.**	JWH-193	95.17	93.37	94.10
**91.**	JWH-198	97.17	94.10	96.73
**92.**	JWH-200	95.83	92.60	94.11
**93.**	JWH-201	91.33	91.50	89.89
**94.**	JWH-213	92.50	89.60	88.71
**95.**	JWH-307	92.17	90.70	89.63
**96.**	JWH-309	88.50	90.03	88.01
**97.**	JWH-368	89.33	84.40	86.42
**98.**	JWH-369	94.00	91.97	88.51
**99.**	JWH-412	91.83	90.10	88.04
**100.**	JWH-424	90.33	89.70	87.61
**101.**	MAM-2201	94.67	94.53	89.88
**102.**	MDMB-CHMICA (MMB-CHMINACA)	88.33	86.40	91.21

No.	COMPOUND	VOLUME OF EXTRACTANT [mL]		
		1	5	10
		RECOVERY [%]		
**103.**	MDMB-CHMINACA	91.00	88.10	88.10
**104.**	MEPIRAPIM	117.00	119.20	105.68
**105.**	MMB018	91.50	89.23	91.19
**106.**	MMB2201	92.17	90.13	90.12
**107.**	MN-18	94.17	88.97	88.92
**108.**	MN-25	92.67	89.10	91.60
**109.**	MN-25-2-METHYL DERIVATIVE	93.33	90.63	90.87
**110.**	MO-CHMINACA	94.50	90.33	89.13
**111.**	NM2201	93.00	90.33	91.85
**112.**	NNEI	91.83	88.83	89.99
**113.**	ORG-28611	113.00	106.73	97.81
**114.**	PB-22	92.50	88.33	92.24
**115.**	PSB-SB-1202	90.67	87.03	89.10
**116.**	PTI-1	110.67	111.00	103.75
**117.**	PTI-2	106.50	107.73	101.45
**118.**	PX-1	94.67	92.10	93.58
**119.**	PX-2	92.67	91.37	92.90
**120.**	RCS-4	90.00	91.03	89.98
**121.**	SDB-005	87.67	89.40	91.68
**122.**	SDB-006	92.67	89.97	90.99
**123.**	THJ	88.50	87.53	90.56
**124.**	THJ-018	91.67	88.90	87.27
**125.**	THJ-2201 (5-FLUORO THJ-018)	97.50	92.23	90.26
**126.**	UR-144	94.33	89.10	90.26
**127.**	WIN 54.461	95.83	92.53	92.93

No.	COMPOUND	VOLUME OF EXTRACTANT [mL]		
		1	5	10
		RECOVERY [%]		
**128.**	WIN 55.212-2	94.00	91.10	88.86
**129.**	XLR-11	91.00	91.37	91.41
**130.**	XLR-12	93.17	91.57	91.17

Table S3. The extraction time and compound's recovery.

**No.**	**COMPOUND**	**EXTRACTION TIME [min]**		
		**10**	**20**	**30**
		**RECOVERY [%]**		
**1.**	Δ-9-TETRAHYDROCANNABINOL (THC)	103.37	93.30	85.16
**2.**	Δ-9-TETRAHYDROCANNABINOL ACID (THCA-A)	113.70	90.43	82.11
**3.**	CANNABIDIOL (CBD)	103.53	90.27	82.42
**4.**	CANNABIDIOLIC ACID (CBDA)	99.87	89.30	83.34
**5.**	CANNABIGEROL (CBG)	92.53	93.00	94.87
**6.**	CANNABIGEROLIC ACID (CBGA)	94.87	91.90	92.35
**7.**	CANNABICHROMENE (CBC)	91.87	91.17	88.92
**8.**	CANNABIVARIN (CBV)	93.03	90.77	92.85
**9.**	CANNABIDIVARINIC ACID (CBDVA)	93.37	88.53	91.84
**10.**	CANNABIGEROVARINIC ACID (CBGVA)	94.53	92.27	89.68
**11.**	CANNABINOL (CBN)	96.53	93.33	93.31
**12.**	TETRAHYDROCANNABIVARINIC ACID (THCVA)	94.37	89.33	91.51
**13.**	TETRAHYDROCANNABIVARIN (THCV)	93.20	91.83	91.52
**14.**	3-CAF	93.53	93.67	93.48
**15.**	5-CHLORO AB-PINACA	93.37	90.43	90.67
**16.**	5-CHLORO-NNEI	94.87	90.13	92.21

No.	COMPOUND	EXTRACTION TIME [min]		
		10	20	30
		RECOVERY [%]		
**17.**	5F-ABICA	116.37	111.97	101.83
**18.**	5F-AB-PINACA	94.03	94.23	93.37
**19.**	5F-ADB (5F-MDMB-PINACA)	92.03	88.50	91.74
**20.**	5F-AMB (5F-AMB-PINACA)	94.03	91.23	91.01
**21.**	5F-APICA (STS-135)	87.03	89.00	91.21
**22.**	5F-APINACA (AKB-48-5F)	95.87	92.20	90.52
**23.**	5-F-JWH-018 ADAMANTYL ANALOG	92.87	90.70	91.20
**24.**	5-FLUORO MN-24 (5-FLUORO NNEI)	96.37	94.37	93.54
**25.**	5-FLUORO THJ	93.20	90.40	91.20
**26.**	5-FLUORO-2-ADB-PINACA ISOMER 2	93.87	91.20	91.97
**27.**	5-FLUORO-CUMYL-PICA	92.37	89.57	88.96
**28.**	5-FLUORO-MN-18	92.20	87.80	88.54
**29.**	5-FLUORO-MN-21 (5-FLUORO-PCN)	94.37	91.33	91.04
**30.**	5-FLUORO-SDB-006	95.87	93.77	94.30
**31.**	5F-PB-22	92.03	91.90	90.09
**32.**	5-F-PENTYL-3-PYRIDINOYLINDOLE	89.20	90.43	88.21
**33.**	5F-SDB-005	92.53	90.50	88.24
**34.**	A-796260	89.03	86.80	91.41
**35.**	A-834735	92.20	89.63	91.39
**36.**	A-836339	93.37	89.50	91.80
**37.**	AB-001	93.70	90.73	92.05
**38.**	AB005	93.20	88.73	92.44
**39.**	AB-CHMINACA	107.20	108.13	101.65
**40.**	AB-FUBINACA	90.70	91.43	90.18
**41.**	AB-FUBINACA 2-FLUOROBENZYL ISOMER	89.20	87.93	90.76

No.	COMPOUND	EXTRACTION TIME [min]		
		10	20	30
		RECOVERY [%]		
**42.**	AB-FUBINACA 3-FLUOROBENZYL ISOMER	95.03	89.50	90.46
**43.**	AB-PINACA	91.70	91.77	91.61
**44.**	ADB-FUBINACA	89.03	85.57	80.72
**45.**	ADB-PINACA	96.20	89.87	84.80
**46.**	ADB-PINACA ISOMER 1	94.53	92.00	86.26
**47.**	ADB-PINACA ISOMER 2	93.87	92.50	85.88
**48.**	ADB-PINACA ISOMER 3	89.03	91.73	93.27
**49.**	ADB-PINACA ISOMER 4	93.87	92.90	92.39
**50.**	AKB48_N-(4-FLUOROBENZYL) ANALOG	93.20	91.27	89.31
**51.**	AM1220	91.53	86.20	89.05
**52.**	AM-1248	92.70	91.00	93.80
**53.**	AM-2201	105.03	102.37	95.17
**54.**	AM2201 8-QUINOLINYL CARBOXAMIDE	94.70	93.07	93.09
**55.**	AM2201 BENZIMIDAZOLE ANALOG	92.03	89.37	92.11
**56.**	AM2232	116.03	106.27	100.95
**57.**	BB-22	94.37	92.50	94.42
**58.**	CB-13	94.53	91.27	91.94
**59.**	CBL-018	93.20	91.63	91.50
**60.**	CUMYL-PICA	90.53	86.67	85.22
**61.**	EAM-2201	93.20	91.17	91.39
**62.**	EG-2201	93.03	92.53	90.98
**63.**	FDU-PB-22	92.20	91.03	90.02
**64.**	FUB-144	87.70	88.53	84.16
**65.**	FUB-JWH-018	92.87	91.47	89.67
**66.**	FUB-PB-22	92.20	91.07	90.43

No.	COMPOUND	EXTRACTION TIME [min]		
		10	20	30
		RECOVERY [%]		
**67.**	HU-210	93.20	88.07	89.38
**68.**	JWH 018 BENZIMIDAZOLE ANALOG	97.03	91.73	91.98
**69.**	JWH-011	92.37	91.37	91.14
**70.**	JWH-016	93.20	91.40	87.86
**71.**	JWH-018	91.03	89.33	88.27
**72.**	JWH-018 ADAMANTYL CARBOXAMIDE	89.87	86.93	91.81
**73.**	JWH-020	93.53	93.10	92.58
**74.**	JWH-022	96.53	93.00	94.31
**75.**	JWH-031	92.87	91.10	89.83
**76.**	JWH-071	94.70	92.37	88.71
**77.**	JWH-073	95.37	94.93	90.08
**78.**	JWH-080	117.70	119.60	105.88
**79.**	JWH-081	94.87	89.37	89.12
**80.**	JWH-098	95.20	90.73	89.33
**81.**	JWH-116	113.70	107.13	98.01
**82.**	JWH-122	111.37	111.40	103.95
**83.**	JWH-145	93.37	91.77	93.10
**84.**	JWH-146	93.37	90.37	91.19
**85.**	JWH-147	98.20	92.63	90.46
**86.**	JWH-149	94.70	91.50	89.06
**87.**	JWH-167	113.37	87.10	81.38
**88.**	JWH-175	91.87	81.00	81.21
**89.**	JWH-182	104.53	87.57	82.14
**90.**	JWH-193	100.87	89.60	81.83
**91.**	JWH-198	100.37	89.77	83.37

No.	COMPOUND	EXTRACTION TIME [min]		
		10	20	30
		RECOVERY [%]		
**92.**	JWH-200	92.87	91.47	92.09
**93.**	JWH-201	92.87	90.07	90.91
**94.**	JWH-213	92.03	90.90	88.27
**95.**	JWH-307	104.03	91.60	88.40
**96.**	JWH-309	93.53	92.43	91.38
**97.**	JWH-368	93.87	91.10	93.16
**98.**	JWH-369	98.53	93.87	94.18
**99.**	JWH-412	93.37	90.33	91.82
**100.**	JWH-424	86.70	94.47	94.81
**101.**	MAM-2201	94.70	92.07	89.63
**102.**	MDMB-CHMICA (MMB-CHMINACA)	93.03	91.13	90.70
**103.**	MDMB-CHMINACA	94.03	90.17	90.79
**104.**	MEPIRAPIM	113.37	104.77	98.79
**105.**	MMB018	91.87	91.23	91.35
**106.**	MMB2201	90.03	88.50	87.08
**107.**	MN-18	93.03	91.27	89.10
**108.**	MN-25	90.70	89.70	90.57
**109.**	MN-25-2-METHYL DERIVATIVE	91.20	85.17	90.17
**110.**	MO-CHMINACA	93.20	88.93	90.23
**111.**	NM2201	96.37	90.90	89.65
**112.**	NNEI	94.37	91.10	90.33
**113.**	ORG-28611	93.03	91.23	89.48
**114.**	PB-22	92.03	89.23	86.97
**115.**	PSB-SB-1202	92.37	89.20	86.40
**116.**	PTI-1	104.20	100.70	88.71

No.	COMPOUND	EXTRACTION TIME [min]		
		10	20	30
		RECOVERY [%]		
**117.**	PTI-2	97.87	94.50	96.93
**118.**	PX-1	93.20	90.00	88.91
**119.**	PX-2	90.03	84.80	86.62
**120.**	RCS-4	91.03	90.10	87.81
**121.**	SDB-005	91.70	88.50	88.30
**122.**	SDB-006	92.87	90.53	90.32
**123.**	THJ	94.03	91.03	91.07
**124.**	THJ-018	92.53	89.23	90.19
**125.**	THJ-2201 (5-FLUORO THJ-018)	91.37	87.43	89.30
**126.**	UR-144	95.37	92.50	93.78
**127.**	WIN 54.461	88.37	89.80	91.88
**128.**	WIN 55.212-2	92.37	89.30	87.47
**129.**	XLR-11	96.53	92.93	93.13
**130.**	XLR-12	93.87	91.97	91.37

Table S4. Summary validation results.

COMPOUND	0.1 ng/mL – 0.01% m/m			0.5 ng/mL – 0.05% m/m			5 ng/mL – 0.5% m/m			50 ng/mL - 5% m/m		
	%CV	%BIAS	U [%]	%CV	%BIAS	U [%]	%CV	%BIAS	U [%]	%CV	%BIAS	U [%]
DELTA-9-TETRAHYDROCANNABINOL (THC)	5.43	12.67	27.57	6.02	-13.30	29.20	3.97	-18.82	38.47	2.70	-10.82	22.30
DELTA-9-TETRAHYDROCANNABINOL ACID (THCA-A)	6.35	-11.67	26.57	5.37	-14.83	31.55	1.77	-19.48	39.12	4.16	-16.42	33.88
CANNABIDIOL (CBD)	6.48	2.67	14.02	6.34	-7.10	19.03	4.10	-15.04	31.17	4.62	-19.37	39.83
CANNABIDIOLIC ACID (CBDA)	5.70	-8.83	21.02	3.26	-19.40	39.34	6.12	-18.99	39.90	5.63	-13.23	28.76
CANNABIGEROL (CBG)	3.43	-4.50	11.31	8.25	-10.53	26.76	3.32	-15.40	31.51	4.56	-18.35	37.82
CANNABIGEROLIC ACID (CBGA)	4.68	13.00	27.64	11.08	-9.97	29.80	4.88	-18.09	37.47	5.75	-7.93	19.59
CANNABICHROMENE (CBC)	9.77	3.83	21.00	5.71	-12.83	28.09	3.75	-18.06	36.88	4.84	-10.91	23.86
CANNABIVARIN (CBV)	13.40	-6.17	29.51	5.74	-8.40	20.35	4.17	-13.94	29.10	3.26	-11.98	24.83
CANNABIDIVARINIC ACID (CBDVA)	10.04	2.83	20.86	5.73	-10.13	23.28	2.68	-17.78	35.96	3.52	-10.73	22.58
CANNABIGEROVARINIC ACID (CBGVA)	7.61	0.17	15.23	8.24	-10.80	27.17	4.14	-18.37	37.66	4.88	-13.10	27.95
CANNABINOL (CBN)	15.19	-6.83	33.30	5.73	-7.90	19.52	3.44	-14.32	29.45	4.39	-13.41	28.21
TETRAHYDROCANNABIVARINIC ACID (THCVA)	6.61	-0.83	13.32	8.87	-11.10	28.42	3.91	-16.86	34.62	3.80	-12.11	25.39
TETRAHYDROCANNABIVARIN (THCV)	11.45	-0.33	22.91	5.39	-10.63	23.84	4.38	-16.83	34.79	6.76	-15.03	32.96
3-CAF	4.03	-11.67	24.69	5.97	-8.67	21.05	4.13	-6.93	16.14	-	-	-
5-CHLORO AB-PINACA	4.32	-8.17	18.48	4.71	-7.40	17.54	3.22	-5.33	12.46	-	-	-
5-CHLORO-NNEI	3.85	-7.83	17.45	6.09	-8.93	21.62	3.74	-8.11	17.86	-	-	-
5F-ABICA	4.67	-6.83	16.56	4.88	-7.50	17.90	3.76	-7.81	17.34	-	-	-
5F-AB-PINACA	3.52	-5.83	13.62	4.92	-8.50	19.65	5.03	-7.85	18.64	-	-	-
5F-ADB (5F-MDMB-PINACA)	3.72	-7.83	17.35	4.68	-10.33	22.69	2.55	-9.29	19.27	-	-	-
5F-AMB (5F-AMB-PINACA)	4.14	-7.50	17.14	5.62	-9.13	21.45	3.79	-10.89	23.06	-	-	-
5F-APICA (STS-135)	4.13	-8.83	19.50	4.28	-9.23	20.35	3.84	-11.28	23.83	-	-	-
5F-APINACA (AKB-48-5F)	4.78	-8.67	19.80	6.00	-9.50	22.47	6.07	-11.93	26.77	-	-	-
5-F-JWH-018 ADAMANTYL ANALOG	4.14	-9.17	20.12	5.88	-14.20	30.74	4.37	-11.15	23.95	-	-	-
5-FLUORO MN-24 (5-FLUORO NNEI)	3.19	-7.67	16.61	5.42	-9.63	22.11	4.93	-7.35	17.70	-	-	-
5-FLUORO THJ	9.67	3.33	20.46	17.10	-8.80	38.46	8.35	-11.80	28.91	-	-	-
5-FLUORO-2-ADB-PINACA ISOMER 2	4.01	-8.00	17.90	4.71	-9.40	21.03	3.81	-6.40	14.91	-	-	-
5-FLUORO-CUMYL-PICA	3.78	-7.33	16.50	3.65	-11.87	24.83	5.20	-8.36	19.70	-	-	-
5-FLUORO-MN-18	3.82	-7.17	16.24	5.44	-7.97	19.30	3.58	-8.82	19.03	-	-	-
5-FLUORO-MN-21 (5-FLUORO-PCN)	2.69	4.33	10.20	3.13	1.97	7.40	3.14	-5.03	11.85	-	-	-
5-FLUORO-SDB-006	4.23	-6.17	14.96	5.36	-8.13	19.48	3.73	-10.52	22.32	-	-	-
5F-PB-22	3.42	-6.83	15.28	5.64	-9.30	21.75	4.69	-7.04	16.91	-	-	-
5-F-PENTYL-3-PYRIDINOYLINDOLE	5.30	-6.00	16.01	4.82	-7.33	17.55	3.93	-7.11	16.25	-	-	-
5F-SDB-005	6.34	-4.17	15.18	7.31	-7.07	20.34	3.61	-6.89	15.56	-	-	-
A-796260	4.31	-2.17	9.65	4.18	-6.53	15.51	3.52	-6.02	13.95	-	-	-

A-834735	3.22	-8.67	18.49	4.03	-11.03	23.49	3.47	-8.09	17.60	-	-	-
A-836339	3.62	-6.33	14.59	4.67	-11.07	24.02	4.71	-8.69	19.77	-	-	-
AB-001	4.71	-7.33	17.43	4.98	-10.07	22.46	3.68	-8.38	18.29	-	-	-
AB005	2.30	15.33	31.01	3.22	5.87	13.38	2.10	0.75	4.46	-	-	-
AB-CHMINACA	3.60	-7.50	16.64	4.99	-8.57	19.82	4.52	-8.68	19.57	-	-	-
AB-FUBINACA	12.20	-14.00	37.13	3.12	-5.93	13.40	4.72	-5.39	14.33	-	-	-
AB-FUBINACA 2-FLUOROBENZYL ISOMER	3.68	-6.33	14.65	4.91	-7.90	18.61	2.91	-5.78	12.94	-	-	-
AB-FUBINACA 3-FLUOROBENZYL ISOMER	4.93	-7.17	17.40	4.93	-6.73	16.69	3.25	-6.72	14.93	-	-	-
AB-PINACA	5.98	-6.00	16.94	4.27	-8.33	18.73	3.50	-10.57	22.28	-	-	-
ADB-FUBINACA	2.18	-6.17	13.08	5.22	-9.13	21.04	3.57	-8.26	18.00	-	-	-
ADB-PINACA	2.95	-7.33	15.81	4.83	-9.97	22.15	3.48	-9.53	20.30	-	-	-
ADB-PINACA ISOMER 1	3.60	-7.67	16.94	5.43	-9.27	21.48	5.28	-9.50	21.74	-	-	-
ADB-PINACA ISOMER 2	1.90	-7.50	15.48	4.46	-8.77	19.67	3.46	-8.70	18.72	-	-	-
ADB-PINACA ISOMER 3	2.64	-5.83	12.80	5.26	-10.27	23.07	3.46	-7.99	17.42	-	-	-
ADB-PINACA ISOMER 4	2.93	-6.67	14.56	5.11	-10.23	22.88	3.06	-9.41	19.80	-	-	-
AKB48_N-(4-FLUOROBENZYL) ANALOG	4.64	-10.17	22.35	4.90	-13.73	29.17	4.58	-14.98	31.33	-	-	-
AM1220	2.82	15.67	31.84	3.09	11.57	23.94	2.78	1.63	6.45	-	-	-
AM-1248	2.84	12.67	25.96	3.27	4.37	10.91	1.96	-1.41	4.83	-	-	-
AM-2201	2.96	-7.50	16.13	4.63	-9.23	20.66	4.09	-8.81	19.43	-	-	-
AM2201 8-QUINOLINYL CARBOXAMIDE	5.41	-6.67	17.17	7.09	-6.17	18.80	4.24	-6.83	16.07	-	-	-
AM2201 BENZIMIDAZOLE ANALOG	3.21	-8.83	18.80	4.85	-9.17	20.74	2.92	-8.85	18.63	-	-	-
AM2232	4.92	-7.67	18.22	5.18	-7.87	18.84	3.66	-9.22	19.84	-	-	-
BB-22	2.99	-8.67	18.34	6.12	-11.90	26.77	4.55	-8.46	19.21	-	-	-
CB-13	3.52	-10.67	22.46	4.76	-11.90	25.63	4.19	-13.12	27.55	-	-	-
CBL-018	5.81	-8.50	20.60	7.01	-9.37	23.40	3.63	-10.18	21.62	-	-	-
CUMYL-PICA	3.37	-6.67	14.94	5.81	-9.17	21.71	5.12	-9.19	21.04	-	-	-
EAM-2201	3.19	-7.67	16.61	4.96	-9.13	20.78	4.54	-11.10	23.98	-	-	-
EG-2201	15.09	-13.00	39.84	6.29	-11.87	26.86	4.51	-16.04	33.33	-	-	-
FDU-PB-22	3.78	-13.67	28.36	5.19	-11.40	25.05	3.06	-8.99	18.98	-	-	-
FUB-144	3.58	-10.00	21.25	4.46	-10.70	23.18	4.35	-9.63	21.13	-	-	-
FUB-JWH-018	3.25	-7.83	16.96	4.99	-8.93	20.47	2.90	-10.53	21.84	-	-	-
FUB-PB-22	3.60	-4.83	12.06	4.43	-8.20	18.64	2.37	-9.68	19.93	-	-	-
HU-210	10.78	-9.50	28.73	8.79	-15.23	35.18	3.74	-10.03	21.41	-	-	-
JWH 018 BENZIMIDAZOLE ANALOG	3.89	-8.50	18.70	4.93	-9.33	21.11	5.44	-9.77	22.36	-	-	-
JWH-011	3.02	-7.83	16.79	5.47	-9.70	22.28	4.86	-9.00	20.45	-	-	-
JWH-016	4.67	-7.50	17.68	6.19	-11.47	26.06	5.97	-9.97	23.23	-	-	-
JWH-018	4.14	-7.50	17.14	4.95	-12.33	26.58	4.46	-10.82	23.40	-	-	-
JWH-018 ADAMANTYL CARBOXAMIDE	5.86	-4.33	14.58	6.24	-6.03	17.36	4.36	-6.66	15.93	-	-	-
JWH-020	9.03	-4.33	20.04	6.22	-9.50	22.71	5.25	-10.55	23.57	-	-	-
JWH-022	2.24	-3.67	8.60	6.54	-8.67	21.72	4.50	-8.22	18.75	-	-	-
JWH-031	4.14	-7.50	17.14	5.41	-10.00	22.73	4.70	-9.00	20.30	-	-	-
JWH-071	4.20	-6.33	15.20	5.62	-9.30	21.73	3.53	-9.87	20.97	-	-	-
JWH-073	4.86	-8.33	19.30	6.78	-9.03	22.59	3.79	-9.06	19.64	-	-	-
JWH-080	4.47	-6.83	16.33	5.16	-9.20	21.10	4.84	-8.23	19.10	-	-	-
JWH-081	4.03	-7.67	17.33	5.91	-9.17	21.81	3.28	-10.72	22.43	-	-	-
JWH-098	3.54	-7.50	16.58	4.13	-9.00	19.80	4.11	-12.34	26.01	-	-	-

JWH-116	4.18	-8.33	18.64	5.98	-10.83	24.75	3.27	-11.24	23.41	-	-	-
JWH-122	5.44	-8.67	20.46	4.24	-11.17	23.89	2.95	-13.23	27.12	-	-	-
JWH-145	2.59	-9.67	20.01	5.13	-11.07	24.39	3.70	-11.93	24.98	-	-	-
JWH-146	3.89	-8.50	18.70	5.73	-12.60	27.68	3.37	-11.66	24.27	-	-	-
JWH-147	4.06	-8.33	18.54	3.89	-11.20	23.71	4.57	-13.80	29.08	-	-	-
JWH-149	3.91	-10.83	23.04	6.02	-13.47	29.51	2.64	-8.39	17.60	-	-	-
JWH-167	3.42	-6.33	14.40	6.18	-9.07	21.95	4.98	-9.16	20.86	-	-	-
JWH-175	8.66	3.50	18.68	12.09	0.30	24.18	5.64	-11.49	25.59	-	-	-
JWH-182	4.59	-7.17	17.02	4.76	-7.30	17.43	3.40	-7.62	16.68	-	-	-
JWH-193	3.96	-4.83	12.49	4.19	-6.63	15.69	3.56	-5.90	13.79	-	-	-
JWH-198	5.14	-2.83	11.74	6.11	-5.90	16.99	2.55	-3.27	8.29	-	-	-
JWH-200	3.33	-4.17	10.66	4.46	-7.40	17.28	3.21	-5.89	13.43	-	-	-
JWH-201	4.88	-8.67	19.89	5.08	-8.50	19.81	3.83	-10.11	21.63	-	-	-
JWH-213	4.03	-7.50	17.03	3.92	-10.40	22.23	3.54	-11.29	23.67	-	-	-
JWH-307	3.46	-7.83	17.13	5.88	-9.30	22.00	4.12	-10.37	22.31	-	-	-
JWH-309	3.70	-11.50	24.16	4.52	-9.97	21.89	3.17	-11.99	24.80	-	-	-
JWH-368	3.72	-10.67	22.60	5.17	-15.60	32.87	4.64	-13.58	28.71	-	-	-
JWH-369	6.42	-6.00	17.57	6.28	-8.03	20.39	2.84	-11.49	23.67	-	-	-
JWH-412	3.67	-8.17	17.91	5.48	-9.90	22.63	2.94	-11.96	24.63	-	-	-
JWH-424	4.98	-9.67	21.75	3.59	-10.30	21.81	4.42	-12.39	26.31	-	-	-
MAM-2201	5.37	-5.33	15.14	4.31	-5.47	13.93	3.65	-10.12	21.51	-	-	-
MDMB-CHMICA (MMB-CHMINACA)	3.77	-11.67	24.52	7.57	-13.60	31.13	3.66	-8.79	19.04	-	-	-
MDMB-CHMINACA	4.23	-9.00	19.89	4.03	-11.90	25.13	4.47	-11.90	25.42	-	-	-
MEPIRAPIM	3.29	17.00	34.63	1.82	19.20	38.57	2.50	5.68	12.40	-	-	-
MMB018	2.99	-8.50	18.02	4.29	-10.77	23.18	4.37	-8.81	19..67	-	-	-
MMB2201	3.18	-7.83	16.91	5.35	-9.87	22.44	4.08	-9.88	21.39	-	-	-
MN-18	4.91	-5.83	15.25	5.00	-11.03	24.23	5.77	-11.08	24.99	-	-	-
MN-25	3.72	-7.33	16.44	5.43	-10.90	24.36	4.15	-8.40	18.75	-	-	-
MN-25-2-METHYL DERIVATIVE	3.93	-6.67	15.48	6.11	-9.37	22.36	3.52	-9.13	19.56	-	-	-
MO-CHMINACA	2.48	-5.50	12.07	4.59	-9.67	21.40	5.95	-10.87	24.78	-	-	-
NM2201	5.44	-7.00	17.73	6.96	-9.67	23.83	4.11	-8.15	18.25	-	-	-
NNEI	2.87	-8.17	17.32	5.19	-11.17	24.62	2.86	-10.01	20.81	-	-	-
ORG-28611	3.96	13.00	27.18	3.09	6.73	14.82	2.82	-2.19	7.14	-	-	-
PB-22	6.73	-7.50	20.15	6.48	-11.67	26.70	5.28	-7.76	18.78	-	-	-
PSB-SB-1202	2.93	-9.33	19.57	5.38	-12.97	28.08	4.10	-10.90	23.28	-	-	-
PTI-1	12.93	10.67	33.52	5.69	11.00	24.77	2.18	3.75	8.68	-	-	-
PTI-2	7.44	6.50	19.75	3.78	7.73	17.22	1.79	1.45	4.61	-	-	-
PX-1	4.36	-5.33	13.78	4.50	-7.90	18.18	3.36	-6.42	14.49	-	-	-
PX-2	2.95	-7.33	15.81	6.54	-8.63	21.66	3.11	-7.10	15.50	-	-	-
RCS-4	5.49	-10.00	22.81	5.26	-8.97	20.80	4.95	-10.02	22.35	-	-	-
SDB-005	5.98	-12.33	27.41	5.85	-10.60	24.22	4.51	-8.32	18.92	-	-	-
SDB-006	5.40	-7.33	18.22	5.06	-10.03	22.47	3.93	-9.01	19.66	-	-	-
THJ	6.10	-11.50	26.03	7.67	-12.47	29.27	3.12	-9.44	19.88	-	-	-
THJ-018	5.68	-8.33	20.16	5.42	-11.10	24.70	3.92	-12.73	26.63	-	-	-
THJ-2201 (5-FLUORO THJ-018)	4.39	-2.50	10.10	5.85	-7.77	19.45	3.06	-9.74	20.42	-	-	-
UR-144	3.40	-5.67	13.21	5.77	-10.90	24.67	4.67	-9.74	21.60	-	-	-

WIN 54.461	2.23	-4.17	9.45	5.49	-7.47	18.54	3.30	-7.07	15.60	-	-	-
WIN 55.212-2	3.62	-6.00	14.02	4.61	-8.90	20.05	4.28	-11.14	23.87	-	-	-
XLR-11	6.22	-9.00	21.88	4.09	-8.63	19.11	2.21	-8.59	17.75	-	-	-
XLR12	4.87	-6.83	16.78	5.15	-8.43	19.77	4.78	-8.83	20.09	-	-	-

Table S5. Summary validation results of dilution integrity.

COMPOUND	0.1 ng/mL - 0.1% m/m			0.5 ng/mL - 0.5% m/m			5 ng/mL - 5% m/m			50 ng/mL - 50% m/m		
	%CV	%BIAS	U [%]	%CV	%BIAS	U [%]	%CV	%BIAS	U [%]	%CV	%BIAS	U [%]
DELTA-9-TETRAHYDROCANNABINOL (THC)	5.05	14.67	31.02	2.67	-0.40	5.40	2.72	5.68	12.60	1.90	-11.48	23.28
DELTA-9-TETRAHYDROCANNABINOL ACID (THCA-A)	4.04	10.17	21.88	3.23	1.53	7.15	3.30	5.41	12.67	2.80	-9.70	20.19
CANNABIDIOL (CBD)	6.27	17.83	37.81	5.43	8.03	19.40	4.12	3.34	10.61	3.83	-10.48	22.31
CANNABIDIOLIC ACID (CBDA)	8.39	15.67	35.55	8.76	11.17	28.38	5.05	14.44	30.60	2.84	-7.10	15.29
CANNABIGEROL (CBG)	3.68	14.00	28.95	3.31	1.37	7.17	4.17	0.35	8.38	4.34	-12.37	26.22
CANNABIGEROLIC ACID (CBGA)	4.45	17.67	36.44	6.05	8.00	20.06	5.50	13.77	29.66	2.23	-8.95	18.44
CANNABICHROMENE (CBC)	3.98	12.83	26.87	3.67	-1.73	8.11	2.15	1.11	4.83	3.03	-8.71	18.44
CANNABIVARIN (CBV)	7.46	14.83	33.21	6.36	-1.77	13.21	4.97	2.11	10.80	3.12	-9.33	19.68
CANNABIDIVARINIC ACID (CBDVA)	11.13	5.50	24.83	5.03	1.77	10.66	2.35	4.27	9.74	2.98	-10.17	21.20
CANNABIGEROVARINIC ACID (CBGVA)	3.81	14.17	29.34	4.66	5.80	14.88	5.10	7.39	17.96	3.06	-8.01	17.16
CANNABINOL (CBN)	5.95	13.17	28.90	3.70	8.17	17.94	4.62	6.65	16.20	3.21	-9.31	19.68
TETRAHYDROCANNABIVARINIC ACID (THCVA)	9.47	10.50	28.28	7.61	-1.23	15.42	3.73	4.86	12.26	3.77	-9.53	20.50
TETRAHYDROCANNABIVARIN (THCV)	7.61	7.33	21.13	6.26	-0.73	12.60	3.71	0.56	7.50	3.16	-9.05	19.16
